# Current Practices in the Processing, Diagnosis, and Reporting of Endometrial Carcinoma: Results of a Web-based Survey by the International Society of Gynecological Pathologists (ISGyP)

**DOI:** 10.1097/PGP.0000000000000515

**Published:** 2018-12-14

**Authors:** Vinita Parkash, Xavier Matias-Guiu, Esther Oliva, Anais Malpica, W. Glenn McCluggage

**Affiliations:** Department of Pathology and Obstetrics and Gynecology, Yale School of Medicine and the Yale School of Public Health, New Haven, Connecticut (V.P.); Department of Pathology, Hospital University Arnau de Vilanova and University de Bellvitge, Irblleida, Idibell, University of Lleida, Ciberonc, Lleida, Spain (X.M.G.); Department of Pathology, Massachusetts General Hospital and Harvard Medical School, Boston, Massachusetts (E.O.); Department of Pathology, The University of Texas MD Anderson Cancer Center, Houston, Texas (A.M.); Department of Pathology, Belfast Health and Social Care Trust, Belfast, UK (W.G.M.)

**Keywords:** Endometrial carcinoma, Processing, Diagnosis, Reporting, Survey

## Abstract

There have been significant advances in our understanding of the biology and classification of endometrial carcinoma, over the last few years, and the new prediction models proposed for prognostication. To accurately diagnose and stage tumors and apply these prediction models, it is necessary that there be standardized processing of specimens, and a common understanding and usage of the diagnostic terminology of endometrial carcinoma. The International Society of Gynecological Pathologists embarked on an ambitious project to achieve this goal in 2015. An early step in the process was to collect baseline information on existing practices with regard to the processing, diagnosis, and reporting of endometrial carcinomas among the members of the society. This was carried out using a web-based survey comprising 112 questions. The results are presented herein and reveal areas of uniformity but also areas of substantial variation among pathologists. The results of the survey assisted in developing the subsequent recommendations that follow as separate articles in this issue of the journal with regard to processing, diagnosis, and reporting of endometrial carcinomas.

Endometrial carcinoma is the sixth most common malignancy of women worldwide (www.wcrf.org/int/cancer-facts-figures/data-specific-cancers/endometrial-cancer-cancer-lining-womb-statistics). It is the most common gynecologic cancer in the developed world and the second most common in the developing world (www.wcrf.org/int/cancer-facts-figures/data-specific-cancers/endometrial-cancer-cancer-lining-womb-statistics). Significant advances have been made in our understanding of the biology and classification of endometrial carcinoma over the past few years, and it is now expected that the pathologic evaluation of a cancer resection specimen will inform not only on staging parameters, but also on accurate subtyping and the provision of prognostic parameters to accurately direct management 1.

There have been no large-scale studies documenting the usual practices for processing, diagnosis, reporting, and ancillary testing of endometrial carcinomas among gynecologic pathologists. The International Society of Gynecological Pathologists (ISGyP) undertook a survey of its members, to investigate these parameters and gather baseline information, and the results are presented herein.

## MATERIALS AND METHODS

Details of the rationale for the project are presented elsewhere in this issue. A 112-question survey was designed by the 5 members of the steering committee appointed by the Board of Directors of the ISGyP (authors of this paper). The survey was piloted, modified, and approved by the members of the Board of Directors and the education committee of the ISGyP. The approved survey was sent to the membership using the SurveyMonkey platform (www.surveymonkey.com). The series of questions explored current practice and perceptions relating to processing, diagnosing, reporting, and ancillary testing of endometrial carcinoma. The questions varied in format, including some with binary responses (Yes/No or True/False), single-choice responses from a list of possibilities, and multiple possible selections from a list of possibilities. For some questions, respondents were offered the opportunity to expand on their responses.

A link to the survey was e-mailed to all members of the society. Participants were given a 6-wk deadline to complete the survey, with 3 reminders sent over that time period.

Respondents were given the opportunity to identify themselves and provide e-mail addresses.

They were incentivized to undertake the survey by indicating that they would be invited to the upcoming ISGyP consensus conference that was scheduled for Seattle, WA, USA, in March 2016, to coincide with the annual United States and Canadian Academy of Pathology meeting. While demographic information (eg, country of practice) was requested, the responses were broadly analyzed as a single cohort, with some comparisons performed between North American and European pathologists, the 2 regions with the largest cohort of respondents.

## RESULTS

There were 242 respondents to the survey representing 47% of the total society membership, with 221 (91.3%) respondents answering all 112 questions. Respondents came from 30 countries with 51% from North America, 19% from Europe (41% of these from the UK), 11% from Asia and Africa, 6% from Oceania, and 2% from South America. Seventy-six percent of respondents self-identified as academics and 24% as pure gynecologic pathologists. The demographics of the respondents are detailed in Table 1.

**TABLE 1:**
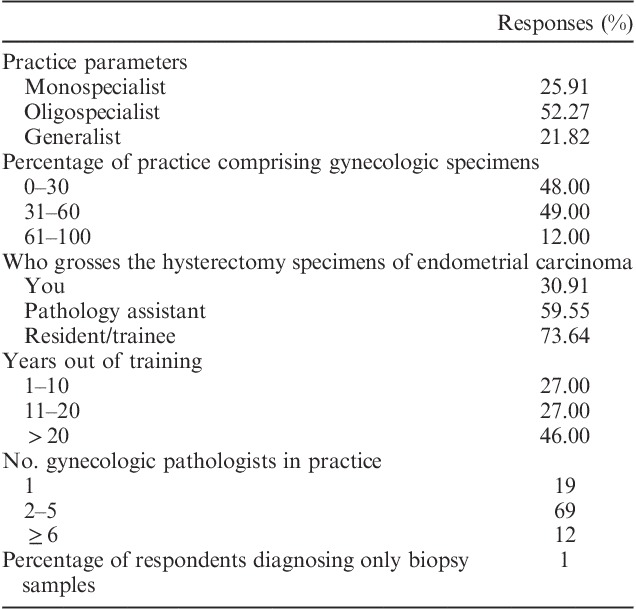
Demographics of respondents to study

Responses with regard to intraoperative assessment (including frozen section examination) are tabulated in Table 2. The responses demonstrated substantial variation among pathologists with regard to the use of intraoperative assessment, with 39% of pathologists never assessing hysterectomy specimens for endometrial carcinoma intraoperatively. Of those who did undertake intraoperative assessment, most indicated that this was carried out either at the surgeon’s request or to assess staging parameters. There was significant variability in the number of sections examined (1–8) and the parameters assessed at intraoperative assessment, with myometrial invasion being the most commonly evaluated (93% of respondents).

**TABLE 2:**
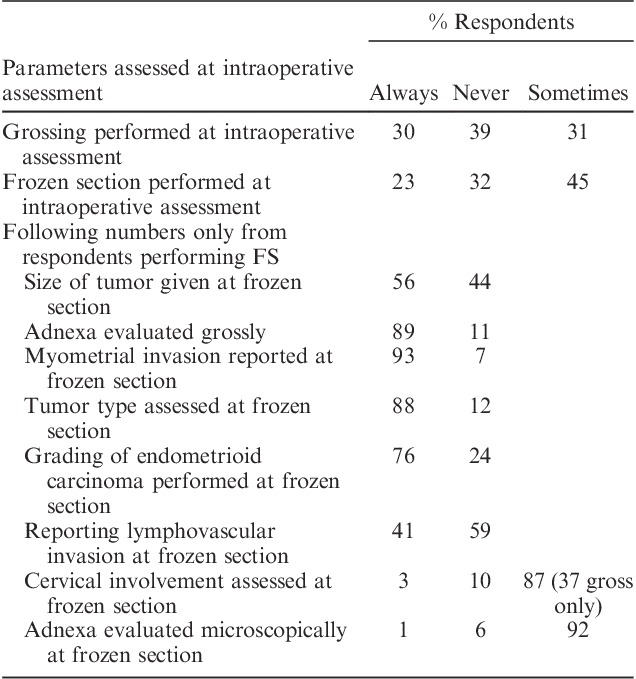
Responses regarding intraoperative assessment

Responses to the grossing practice questions are tabulated in Table 3, and they also revealed significant variability in practice with regard to some parameters. There was broad agreement with respect to several parameters such as recording tumor size and location (99% and 94%, respectively), including fimbrial sections of the fallopian tube for histologic assessment (95%), submission of the entire endometrium in resection specimens for atypical hyperplasia/endometrial intraepithelial neoplasia (92%), and microscopic evaluation of entire lymph nodes (95%). However, there was variability in the number of sections of tumor examined, ranging from 1 to 2 sections per cm of the entire tumor (responses not shown). There was significant disagreement in practice with respect to other parameters, for example, inking of serosa (46%), evaluating a single section from grossly normal ovaries (50%), and lymph node assessment (53% not examining residual fatty tissue and 47% undertaking sentinel node assessment).

**TABLE 3:**
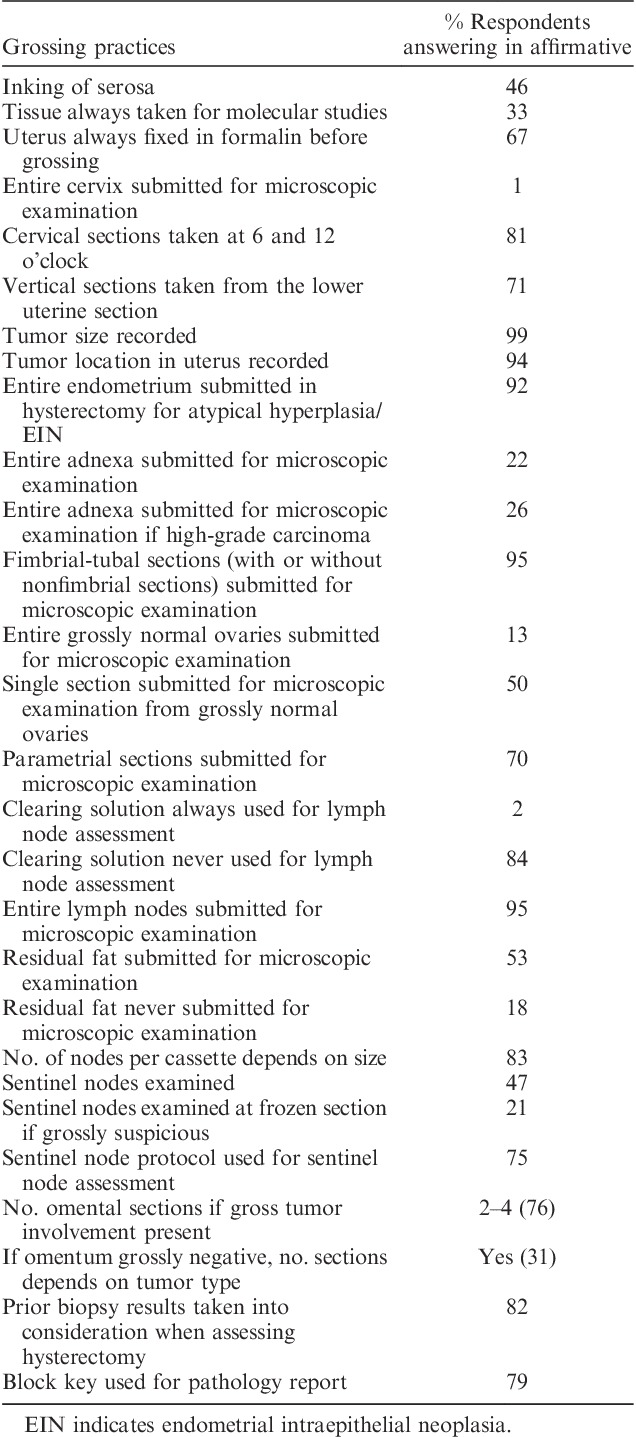
Responses regarding gross assessment of endometrial carcinomas

Microscopic variables assessed and reported are tabulated in Table 4. As a general observation, there was greater variability in the assessment of microscopic than gross parameters. International federation of gynecology and obstetrics (FIGO) grading was used by 97% of respondents. However, there was variability in the more granular use of the FIGO grading system, with 17% of respondents indicating that they assigned mixed FIGO grades to phenotypically heterogenous tumors. Reporting of background endometrium and cervical gland involvement was performed by 92% and 90% of respondents, respectively. There was significant variability with respect to minimal and necessary criteria to diagnose high-grade subtypes of endometrial carcinoma (serous, clear cell, carcinosarcoma, undifferentiated), with 22% to 60% of respondents variably using morphology alone to histotype tumors. Nine percent of respondents indicated that they did not report tumor stage in pathology reports; of those that did, 94% used the FIGO staging system with or without the TNM stage.

**TABLE 4:**
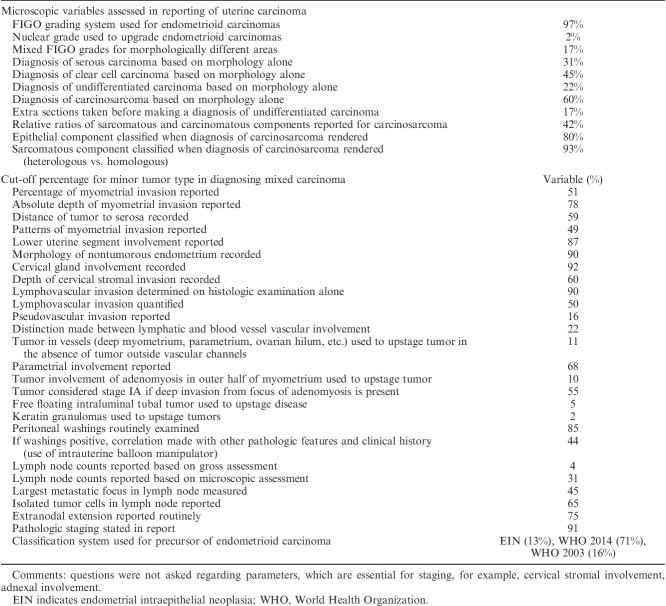
Responses regarding microscopic assessment of endometrial carcinomas

There was also substantial variability in ancillary testing of endometrial carcinomas, although testing for mismatch repair protein abnormalities and possible Lynch syndrome was performed in at least a subset of cases by 82% of respondents (Table 5), with 27% of respondents undertaking the studies in all cases of endometrial carcinoma. Table 6 documents the parameters used to trigger the aforementioned testing.

**TABLE 5:**
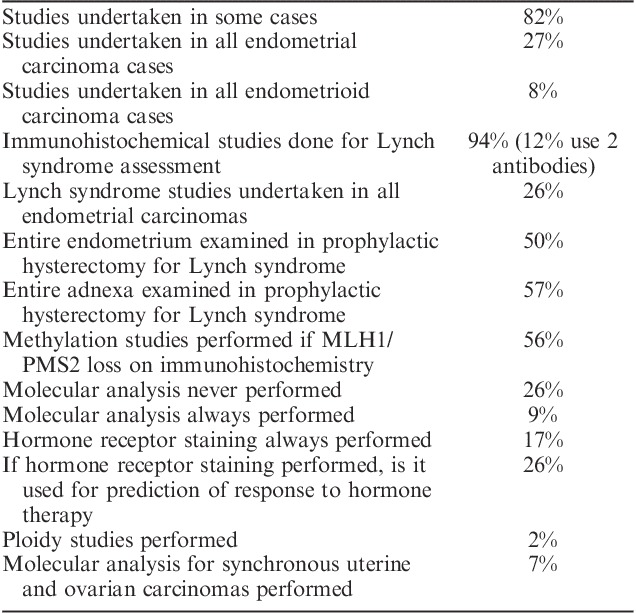
Responses regarding ancillary testing of endometrial carcinomas

**TABLE 6:**
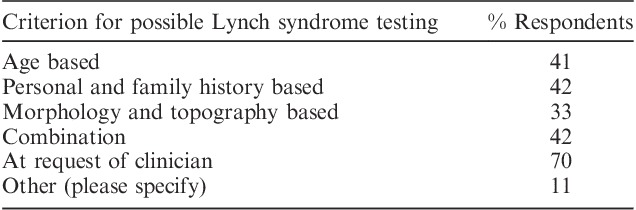
Parameters used to trigger testing for possible Lynch syndrome

Some variability in practice was apparent, between North American and European pathologists, as shown in Table 7. Differences included percentage of pathologists involved in grossing of specimens, use of intraoperative assessment, routine use of immunohistochemistry to histotype tumors, and performing studies to assess possible Lynch syndrome. North American pathologists generally used both TNM and FIGO staging systems, whereas European pathologists favored using only the FIGO system.

**TABLE 7:**
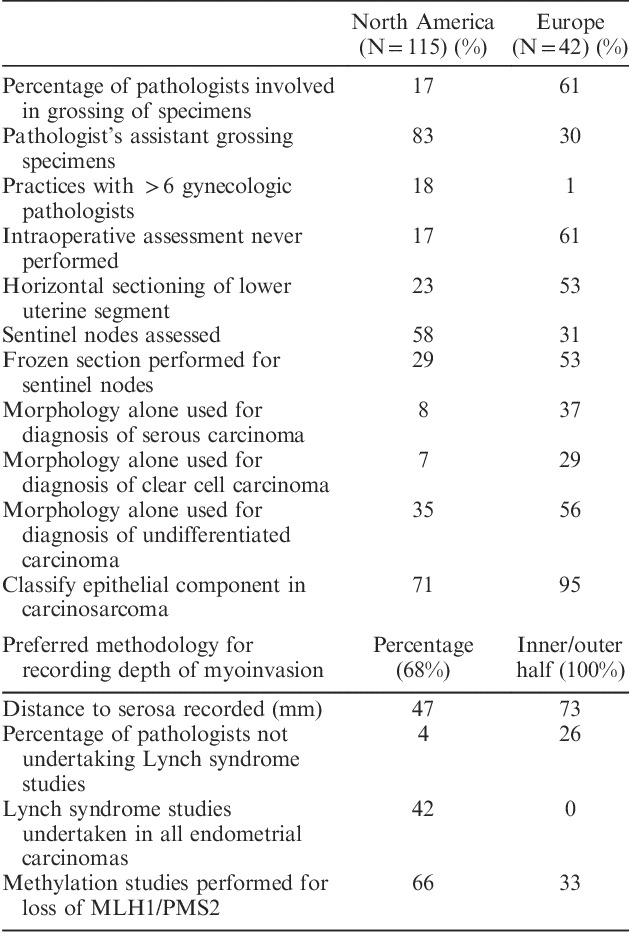
Comparison between North American and European pathologists

## DISCUSSION

Recent years have witnessed an explosive growth in our understanding of endometrial carcinoma 1. Like ovarian carcinoma, endometrial carcinoma is not 1 entity, or even 2, as proposed by Bokhman 2, but comprises at least 4 distinctive molecular subtypes with differing prognoses 1. Another significant development has been the identification of endometrial carcinoma as a common, and often sentinel, tumor in Lynch syndrome, one of the most common hereditary cancer syndromes. These discoveries have led to recommendations from clinical societies for extended reporting of prognostic factors and for routine screening of endometrial carcinomas for mismatch repair protein abnormalities 3. However, the impact of these advances on the practice of specialist gynecologic pathologists around the world is unknown. The ISGyP therefore undertook a web-based survey to document current practice patterns before convening a consensus conference and issuing guidelines for the diagnoses, reporting, and ancillary testing of endometrial carcinoma.

The results of our survey show certain consistent practices among gynecologic pathologists worldwide. For example, at grossing, recording tumor size and site, examination of the entire endometrium in resection specimens for atypical hyperplasia/endometrial intraepithelial neoplasia, and assessment of the fimbrial end of the fallopian tube appear embedded as routine practice. At microscopic assessment, reporting of tumor grade, pathologic stage, documentation of depth of myometrial invasion, assessment of cervical glandular involvement, and lymphovascular space invasion are near universal practices. There is also widespread acceptance and utilization of the FIGO grading and staging systems. That said, there are also areas of significant practice variability, including the number of sections of tumor, adnexal tissue, omentum, and lymph nodes examined. While FIGO grading is used universally for reporting, the actual usage shows some variability, with 17% of respondents using mixed grading for phenotypically heterogenous endometrioid carcinomas. Histotyping of nonendometrioid carcinomas, not unexpectedly, seems to be a particularly challenging area for gynecologic pathologists, with morphologic criteria alone being used for subtyping in 22% to 60% of cases, depending on the favored subtype, with ancillary studies being used variably. This raises some concerns about the consistency of diagnostic criteria across studies and institutions, especially as several studies have shown significant variability in the classification and typing of high-grade endometrial carcinoma, even among expert gynecologic pathologists 4–6.

Similarly, there is substantial variability with respect to assessment of risk for Lynch syndrome, with only a subset of pathologists performing these studies on all cases of endometrial adenocarcinomas (27%). Immunohistochemistry is the favored modality for initial investigation.

Our study also documents some practice differences between North American and European pathologists. In North America, there is a greater use of pathology assistants for grossing of specimens, greater use of intraoperative assessment and frozen sections, and an increased propensity to use ancillary studies for subtyping. North American pathologists are also more likely to report a TNM stage than European pathologists. This is not surprising, as in the United States, use of the American Joint Committee on Cancer version of TNM is required for College of Surgeons Cancer Center accreditation, NCCN Clinical decision guidelines implementation, and for the College of American Pathologists accreditation.

A drawback of this survey is that it was only sent to the members of ISGyP, and, as such, the results are skewed toward the practices of specialist gynecologic pathologists at academic centers. In spite of a relatively high participation rate of 47%, the possibility of a self-selection bias cannot be excluded. As always, reporting of the expected ideal rather than the practiced behavior may have influenced the results.

## CONCLUSIONS

The survey shows areas of concordance and variability in practice among gynecologic pathologists worldwide in dealing with endometrial carcinoma specimens.

The results of this survey helped identify areas in need of consensus to standardize processing, diagnostic and reporting criteria, and ancillary testing of endometrial carcinoma. These results informed the deliberations of the Endometrial Carcinoma Project subcommittees for the International Society of Gynecological Pathology. The remaining articles in this journal describe the process and the recommendations that emerged from that project.
